# Cell–cell adhesion: linking Wnt/β-catenin signaling with partial EMT and stemness traits in tumorigenesis

**DOI:** 10.12688/f1000research.15782.1

**Published:** 2018-09-18

**Authors:** Sayon Basu, Sanith Cheriyamundath, Avri Ben-Ze’ev

**Affiliations:** 1 Department of Molecular Cell Biology, Weizmann Institute of Science, Rehovot, Israel

**Keywords:** Cell-cell adhesion, beta-catenin signaling, tumorigenesis

## Abstract

Changes in cell adhesion and motility are considered key elements in determining the development of invasive and metastatic tumors. Co-opting the epithelial-to-mesenchymal transition (EMT) process, which is known to occur during embryonic development, and the associated changes in cell adhesion properties in cancer cells are considered major routes for tumor progression. More recent *in vivo* studies in tumor tissues and circulating tumor cell clusters suggest a stepwise EMT process rather than an “all-or-none” transition during tumor progression. In this commentary, we addressed the molecular mechanisms underlying the changes in cell adhesion and motility and adhesion-mediated signaling and their relationships to the partial EMT states and the acquisition of stemness traits by cancer cells.

## Introduction

Cell–cell adhesion is a fundamental biological process in multicellular organisms which defines cellular and tissue morphogenesis ^1^. The signaling conveyed by adhesion between cells and with the underlying extracellular matrix (ECM) is tightly coordinated with gene regulation during normal tissue homeostasis ^2, 3^. Disruption of cell–cell adhesion, the subsequent changes in adhesion-mediated signaling, and the increase in cell motility are characteristic steps observed during the development of invasive metastatic cancer. Tumor progression and embryonic development often display the process of epithelial-to-mesenchymal transition (EMT) that results in the conversion of cells with strong cell–cell adhesion and an epithelial morphology into cells with more motile and invasive characteristics, as seen in mesenchymal cells ^4^. EMT includes a weakening of strong intercellular homotypic cadherin-based cell–cell junctions, and of the cadherin–catenin anchorage to the cytoskeleton, while inducing the expression of adhesion molecules that convey weaker and heterotypic adhesion. These properties increase the ability of cancer cells to detach from the primary tissue, bind to ECM components and to other cells (for example, fibroblasts), and migrate toward the lymph and blood systems. In addition to displaying EMT-like properties, invasive tumor cells display traits seen in normal stem cells and therefore are described as cancer stem cells (CSCs) ^5^.

Cell–cell adhesion is regulated by cell adhesion molecules (CAMs), which are transmembrane receptors linked to the cytoskeleton that govern the assembly of cells into three-dimensional tissues ^6^. CAMs also determine the interaction of cells with the surrounding environment and regulate the response to external stimuli. Changes in the expression of and signaling by CAMs play an important role in different stages of cancer development ^6^. Here, we discuss recent studies on how changes in cell adhesion, EMT, and the acquisition of stem cell properties can promote invasive cancer development.

## Cancer-associated epithelial-to-mesenchymal transition is not a binary switch but a stepwise transition process

The process of EMT in cancer cells confers properties that enhance their metastatic ability via increased motility and invasiveness, the ability to dismantle the ECM, and an increased potential to induce a stem cell–like state ^7, 8^. Recent reports suggested that a complete EMT is not essential for metastasis ^9– 13^. Metastatic cancer cells were shown to depend on epithelial cytokeratin expression for survival ^14^, and in a study ^15^ with breast cancer cells, metastasis was shown to require the expression of epithelial cell adhesion (E- and P-cadherin) and cytoskeletal (cytokeratins K5, K8, and K14) proteins ^14– 16^. While the adhesive properties of cells are altered during EMT, a binary switch from an epithelial to a mesenchymal state is rarely seen during normal embryonic development ^11, 17^ or in cancer ^9, 17^. Instead, cells undergoing EMT attain various states that are intermediate between the epithelial origin and a mesenchymal potential, evident by the co-expression of CAMs for both epithelial and mesenchymal states, together with a partial loss of E-cadherin or its displacement from the cell membrane or both ^18, 19^. Such stepwise/partial EMT observed in cancer cells may explain tumor heterogeneity and the different tumorigenic abilities of cancer cells from various niches within the tumor. Cancer cells with a partial EMT were found to be more efficient in tumor budding, invasion, and metastasis since these processes apparently require both EMT and mesenchymal-to-epithelial transition (MET) ^9^.

A partial EMT was described in studies on different tumor types ^20– 26^. In ovarian cancer, the EMT process was subdivided into four types depending on the degree of epithelial or mesenchymal marker expression ^21^. The transforming growth factor-beta (TGF-β)-induced EMT in MCF10A breast epithelial cells is also a stepwise process with intermediary states ^20^. In studies on skin and breast cancer in mouse models and in patient-derived xenograft (PDX) tissues, cancer cells undergoing EMT were subdivided into six phenotypically and functionally distinct states on the basis of the extent of loss of the epithelial marker EpCAM (epithelial cell adhesion molecule) and gain of the cell–ECM adhesion receptor integrin α5 (CD51), integrin β3 (CD61), and vascular cell adhesion molecule 1 (VCAM1) (CD106) ^25^. After intravenous injection, cells displaying a hybrid EMT subtype were better at reaching the circulation, colonizing the lungs, and forming metastases ^25^. Different CAMs that are used to identify stages of stepwise EMT apparently also play a functional role in the increased metastatic ability of such cells ^8^.

## Intermediate epithelial-to-mesenchymal transition states, circulating tumor cell clusters, and metastasis

Studies in recent years revealed that the dissemination of cancer cells occurs as multicellular clusters rather than single cells, and circulating tumor cells (CTCs), which play a key role in determining metastatic ability, also involve an incomplete/intermediate/partial EMT ^19, 27^. CTC clusters were reported in different types of cancer (breast, lung, colorectal, and prostate), and increased CTC clusters were observed during the later stages of metastasis ^28, 29^. Individual cells within the CTC clusters display different EMT subtypes that contribute to successful survival within the circulation, extravasation, and tumor seeding ^19, 24– 26^. Previous studies hypothesized that a single disseminated tumor cell is the precursor of a metastatic tumor. When a cluster of different tumor cells is involved, the metastatic tissue is polyclonal, providing a possible explanation for variations in response to cancer therapy ^27^. In CTC clusters, the cells remain cohesive to each other because of the presence of EpCAMs on their surface ^21, 30^.

In pancreatic ductal adenocarcinoma (PDAC), more differentiated cells with partial EMT traits expressing E-cadherin, claudin-7, and EpCAM were shown to invade and metastasize as multicellular tumor clusters ^26^. In a study with human breast cancer cells, CTC clusters were shown to be more metastatic than single CTCs ^31^. Such CTC clusters displayed an increase in the junctional cytoplasmic plaque protein plakoglobin (γ-catenin) ^31^. Plakoglobin is a paralogue of β-catenin that, in addition to its interaction with adherens junctions, is a key component of desmosomes, a specialized epithelial cell–cell adhesion structure ^32^. When compared with β-catenin (whose overexpression enhances tumorigenesis), plakoglobin is associated mostly with tumor suppression ^33^. Interestingly, breast cancer cells expressing plakoglobin that are shed into the circulation as multicellular clusters were more metastatic than plakoglobin-deficient single cells ^31^. It was suggested that plakoglobin-expressing CTCs are more likely to get trapped in small capillaries than are single tumor cells, thereby promoting the extravasation of CTC clusters in distant organs ^31^.

In inflammatory breast cancer (IBC), CTC clusters that enhance metastasis are organized as intra-lymphatic tumor cell emboli that retain membrane-bound E-cadherin, maintain cell–cell adhesion, and invade as clusters that display a partial EMT (characterized by vimentin expression) ^34^. Invasion via CTC clusters can also involve the activation of α-catenin, another adherens junction plaque protein that promotes the contractility of the actomyosin system in cell–cell junctions ^35^. Cancer cells expressing E-cadherin were recently shown to form heterotypic adhesion complexes with cancer-associated fibroblasts that express N-cadherin, thereby assisting in cancer cell migration ^36^. Such a mechanotransduction process could explain why E-cadherin expression at the surface of CTC clusters increases their invasive ability.

## Epithelial-to-mesenchymal transition, cancer stem cells, and metastasis

A link between cell adhesion and stem cell–like properties in cancer cells was proposed on the basis of serial tumor sections in which cells at the invasive edge of the tumor (that eventually bud off into the circulation and metastasize) displayed EMT markers together with stemness characteristics ^37^. Moreover, in a set of mammary tumor cell lines, the loss of E-cadherin was shown to induce stem cell–like behavior (enrichment in CD44 ^high^/CD24 ^low^ stem-like cells and ability to form mammospheres) ^38^. Other studies in a murine model of mammary tumor revealed that both normal and cancer stem cells display characteristics of EMT ^10^. An increased expression of the EMT transcription factor (EMT-TF) ZEB1 (which inhibits E-cadherin transcription) was reported in tumor-initiating stem-like cells in colorectal and pancreatic cancer ^39^. The stabilization of β-catenin by SNAIL (another EMT-TF) expression leads to the expansion of the stem cell niche in colorectal cancer (CRC) ^40^. Together, these observations suggested that EMT induces stemness in invasive cancer cells. Since the loss of E-cadherin and transition into a mesenchymal state often characterize CSCs, a pharmacologically triggered MET was proposed as a potential therapeutic approach aiming to eliminate tumor-initiating cells ^41^. Indeed, by inducing MET and E-cadherin expression in mammary tumor stem cells (through an increase in cyclic AMP [cAMP] and a consequent activation of protein kinase A, or PKA), a suppression of the tumorigenic potential was observed ^42^.

However, an induction of MET might not always be an effective means to block metastasis, since several studies reported that a partial loss of EMT, or a MET-like process, is often required for successful metastasis ^43– 45^. For example, loss of the EMT-TF Prrx1 in breast cancer cells induces MET and leads to the establishment of a CSC niche and was required for metastasis ^43^. In squamous cell carcinoma (SCC), a Twist-1–mediated EMT was necessary in primary tumor cells for local invasion and the intravasation of tumor cells into the circulation, but the silencing of Twist-1 and the re-acquisition of E-cadherin were necessary for extravasation and colonization in the distant tissue ^44^. TGF-β was shown to trigger MET through activation of the TF inhibitor of differentiation-1 (ID1) ^45^, which leads to re-expression of E-cadherin and the submembrane sequestering of β-catenin, together with the loss of mesenchymal vimentin. Such ID1-induced MET also confers CSC-like characteristics (enrichment of CD44 ^high^/CD24 ^low^ cells and the formation of mammospheres). While an EMT and the loss of E-cadherin are advantageous at the primary tumor site, CSCs in metastatic foci re-express E-cadherin under the influence of TGF-β and ID1. Such observations indicate that the epithelial or mesenchymal state of cancer cells affects their stemness/invasive properties differently, depending on whether the cells are localized in the primary tumor, in the circulation, or at distant metastatic sites. In another study with mammary epithelial cells, long-term constitutive activation of Twist-1 led to the production of non-proliferative migratory cells ^46^, while a transient induction of Twist-1 led to the expression of both epithelial (E-cadherin) and mesenchymal (vimentin) states together with CSC characteristics and a greatly enhanced invasive metastatic phenotype ^46^. Twist-1 levels in skin cancer cells determine the extent of their invasiveness, but both low Twist-1 (low E-cadherin, benign papilloma) and high Twist-1 (no E-cadherin, malignant tumor) cells showed stem cell–like behavior, indicating that stemness traits in cancer cells do not require a complete loss of the epithelial phenotype ^47^. Finally, numerous recent studies also described how cells that display both epithelial and mesenchymal markers are enriched in CSC markers and become more metastatic and how such cells acquire stem cell–like traits before they lose E-cadherin ^22, 25, 26, 46, 48^.

Epithelial or mesenchymal cancer cell adhesion properties could also be involved in determining the metastatic site (organotropism) of tumor cells. In a murine model of PDAC, the stabilization of E-cadherin by the adherens junction plaque protein δ-catenin (p120) was essential for liver metastasis, while cells displaying a full EMT metastasized to the lungs, even in the absence of p120 ^49^. Thus, while EMT at the primary tumor site was associated with an induction of stemness and enhanced invasiveness, it is unclear whether both a partial reversion to an epithelial state and the acquisition of CSC properties are required for successful metastasis.

## Signaling via cell adhesion molecules in cancer stem cells

Studies with EMT-TFs involved in the loss of E-cadherin suggest that changes in cell adhesion properties are required to establish and maintain cancer cell stemness traits ^41^. In both normal and cancer stem cells, the activation of WNT signaling and EMT-associated changes in cell adhesion involve the displacement of β-catenin from adherens junctions, where it links E-cadherin to the actin cytoskeleton ^50^. In most types of cancer, β-catenin accumulates and evades the proteasomal destruction complex, translocates into the nucleus, and induces the transactivation of target genes together with lymphoid enhancer factor/T-cell factor (LEF/TCF) factors ^50^. These target genes often include CAMs that promote the generation of CSCs ^51^.

The CAM CD44 is induced by EMT; it is a β-catenin–TCF target gene and integrates multiple signaling pathways that lead to the development of CSC traits. CD44 anchors cells to the ECM by binding to hyaluronic acid and activates various signaling pathways ^52, 53^. Overexpression of CD44 marks cells with CSC properties and is associated with advanced stages of cancer development in the breast, bone, parathyroid gland, liver, colon, and pancreas ^52^. Loss of E-cadherin in breast cancer cells is associated with increased CD44 expression and stemness ^38^. The accumulation of nuclear β-catenin was shown to increase CD44 expression in normal intestinal epithelial and in CRC cells ^54^. CD44, in turn, enhances WNT/β-catenin signaling by phosphorylating the WNT receptor LRP6 ^55^ and can also act via the JAK/STAT3 pathway in various CSC subpopulations ^56^. In CRC cells, the CD44 adhesion receptor is internalized, forms a complex with STAT3 and p300, translocates into the nucleus, and activates cancer-associated genes ^57^. In nasopharyngeal carcinoma and breast cancer, CD44 activates STAT3 and promotes the maintenance of a stem cell–like subpopulation ^58, 59^. In addition to WNT/β-catenin and JAK/STAT3 signaling, PI3K/AKT signaling is induced by CD44. The generation of breast CSCs can involve a BMP-2–mediated degradation of Rb, leading to SMAD activation and increased CD44 expression ^60^. CD44s and CD44v isoforms are prognostic markers for several cancers, and CD44 is used in targeted cancer therapy using anti-CD44 antibodies and CD44 antagonizing peptides ^61^.

The neural cell adhesion immunoglobin-like molecule L1CAM (L1) is another WNT/β-catenin target gene that induces stemness in cancer cells by activating various signaling pathways ^47^. Increased β-catenin-TCF–mediated transactivation enhances L1 expression in CRC cells, resulting in elevated cell motility, invasiveness, and liver metastasis ^62, 63^. The mechanisms downstream of L1 involve the activation of nuclear factor kappa light chain enhancer of B cells (NF-κB) signaling through the scaffold protein ezrin and the Rho-associated protein kinase, leading to the elevated transcription of IGFBP2, SMOC2, and LGR5, known markers of intestinal and colonic epithelial stem cells ^64, 65^. The L1-mediated induction of NF-κB signaling and the activation of stemness-associated genes support studies demonstrating that NF-κB activation induces dedifferentiation and the establishment of CSCs in the intestinal epithelium ^66^. In both mammary epithelial and CRC cells, L1 can suppress E-cadherin and induce nuclear β-catenin accumulation ^67, 68^. In CRC cells, the L1-mediated increase in β-catenin–TCF transactivation results in increased ASCL2, a TF that determines intestinal stem cell fate by regulating various stemness-associated genes ^69^. The induction of ASCL2 and NF-κB activation by L1 are examples of the means by which changes in cell adhesion during tumorigenesis can induce stemness traits in cancer cells. Therapeutic approaches, including short hairpin RNA/small interfering RNA (shRNA/siRNA)-mediated downregulation of L1 or high-affinity monoclonal antibodies against L1, have shown promise in several different cancers ^70^.

The cell–ECM adhesion receptors of the integrin family represent another means by which changes in cell adhesion can affect CSCs and metastasis. The integrin subunits α6(CD49), β1(CD29), and β3(CD61) are known markers of both normal and cancer stem cells ^71– 75^. For example, the knockdown of integrin α6 in glioblastoma cells severely affects a stem cell subpopulation, inhibiting its self-renewal, proliferation, and tumor formation ^76^. The expression of integrin β3 induces various CSC subpopulations in breast, lung, and pancreatic cancer ^73, 77^. Integrins signal through KRAS, NF-κB, and SRC kinase-mediated activation of EMT TFs in stem cells during normal development ^78^. Therapeutic targeted interference with integrin-mediated signaling, such as the targeting of focal adhesion kinase (FAK) activation or of integrin–ECM interactions through enzymes such as lysyl oxygenase or glycocalyx proteins (that is, mucins), are being investigated for designing cancer therapeutics ^79^.

Another mechanism operating during integrin-induced stemness traits involves integrin-linked kinase (ILK). ILK is a focal adhesion-associated serine/threonine kinase that interacts with several integrins in a complex with Parvin and PINCH and acts as a multifunctional effector of growth factor signaling and cell–ECM interactions ^80, 81^. In CRC, ILK induces β-catenin–LEF1 interaction and transactivation, the loss of E-cadherin via induction of EMT-TFs, and the induction of stem cell markers ^82– 88^. In breast CSCs, interleukin-6 (IL-6), a key growth factor, induces the expression of the TF E2F1 via a STAT3/cyclin D1/CDK2 loop and subsequently triggers the activation of NF-κB and NOTCH signaling, which enhance ILK expression ^89^. Activation of ILK is required for breast CSC maintenance ^90^, and ILK also responds to mechanical stress and hypoxia by activating PI3K/AKT signaling, which leads to the expression of CD44 and other stemness-associated genes in breast cancer cells ^91^.

## Conclusions and perspectives

The development of distant metastases, after the formation of a primary tumor, involves many stages and often takes decades in humans. The molecular basis of the numerous steps involved in the process of metastasis is still poorly understood. While co-opting the EMT process by cancer cells is considered a key step, recent *in vivo* studies revealed that EMT does not proceed by a binary step from an epithelial to a mesenchymal state. Rather, it involves many stages and variations on the theme with different hybrid epithelial/mesenchymal states being the rule rather than the exception. Hybrid epithelial/mesenchymal states, the development of stemness characteristics, and the gain and sometimes loss of the same traits during the long process of metastasis point to the high degree of plasticity in the cancer cell phenotype. The studies discussed here show that there are a number of tumor cell subpopulations in the same tumor, displaying varying degrees of EMT. Which EMT stage is necessary for the induction of stem cell traits and what the molecular signaling steps involved in this process are remain to be determined. The role for and necessity of these different tumor cell subpopulations for successful metastasis also await further investigation. The changes in CAMs and the associated cytoskeletal proteins involved in the trans-differentiation and hybrid EM phenotypes are only starting to be revealed. Careful *in vivo* analyses of human tumors and studies in animal models *in vivo* will hopefully determine the molecular characteristics of the changes in cell adhesion and motility as related to these cancer cell phenotypes and the associated stemness traits and their relevance to the development of metastases and will hopefully provide future avenues for effective cancer therapies.

## Abbreviations

CAM, cell adhesion molecule; CRC, colorectal cancer; CSC, cancer stem cell; CTC, circulating tumor cell; ECM, extracellular matrix; EMT, epithelial-to-mesenchymal transition; EMT-TF, epithelial-to-mesenchymal transition-associated transcription factor; EpCAM, epithelial cell adhesion molecule; ID1, inhibitor of differentiation-1; ILK, integrin-linked kinase; L1 or L1 CAM, L1 cell adhesion molecule; LEF, lymphoid enhancer factor; MET, mesenchymal-to-epithelial transition; NF-κB, nuclear factor kappa light chain enhancer of B cells; PDAC, pancreatic ductal adenocarcinoma; TCF, T-cell factor; TF, transcription factor; TGF-β, transforming growth factor-beta.

## References

[1] Wickström SA Niessen CM: Cell adhesion and mechanics as drivers of tissue organization and differentiation: local cues for large scale organization. *Curr Opin Cell Biol.* 2018;54:89–97. 10.1016/j.ceb.2018.05.003 29864721

[2] Conacci-Sorrell M Zhurinsky J Ben-Ze'ev A: The cadherin-catenin adhesion system in signaling and cancer. *J Clin Invest.* 2002;109(8):987–91. 10.1172/JCI15429 11956233 PMC150951

[3] Clément R Dehapiot B Collinet C: Viscoelastic Dissipation Stabilizes Cell Shape Changes during Tissue Morphogenesis. *Curr Biol.* 2017;27(20):3132–3142.e4. 10.1016/j.cub.2017.09.005 28988857

[4] Nieto MA Huang RY Jackson RA: EMT: 2016. *Cell.* 2016;166(1):21–45. 10.1016/j.cell.2016.06.028 27368099

[5] Polyak K Weinberg RA: Transitions between epithelial and mesenchymal states: acquisition of malignant and stem cell traits. *Nat Rev Cancer.* 2009;9(4):265–73. 10.1038/nrc2620 19262571

[6] Cavallaro U Christofori G: Cell adhesion and signalling by cadherins and Ig-CAMs in cancer. *Nat Rev Cancer.* 2004;4(2):118–32. 10.1038/nrc1276 14964308

[7] Zhang Y Weinberg RA: Epithelial-to-mesenchymal transition in cancer: complexity and opportunities. *Front Med.* 2018;12(4): .361–373. 10.1007/s11684-018-0656-6 30043221 PMC6186394

[8] Brabletz T Kalluri R Nieto MA: EMT in cancer. *Nat Rev Cancer.* 2018;18(2):128–34. 10.1038/nrc.2017.118 29326430

[9] Jolly MK Mani SA Levine H: Hybrid epithelial/mesenchymal phenotype(s): The 'fittest' for metastasis? *Biochim Biophys Acta.* 2018;1870(2):151–157, pii: S0304-419X(18)30064-7. 10.1016/j.bbcan.2018.07.001 29997040

[10] Ye X Tam WL Shibue T: Distinct EMT programs control normal mammary stem cells and tumour-initiating cells. *Nature.* 2015;525(7568):256–60. 10.1038/nature14897 26331542 PMC4764075

[11] Zheng X Carstens JL Kim J: Epithelial-to-mesenchymal transition is dispensable for metastasis but induces chemoresistance in pancreatic cancer. *Nature.* 2015;527(7579):525–30. 10.1038/nature16064 26560028 PMC4849281

[12] Fischer KR Durrans A Lee S: Epithelial-to-mesenchymal transition is not required for lung metastasis but contributes to chemoresistance. *Nature.* 2015;527(7579):472–6. 10.1038/nature15748 26560033 PMC4662610

[13] Aiello NM Brabletz T Kang Y: Upholding a role for EMT in pancreatic cancer metastasis. *Nature.* 2017;547(7661):E7–E8. 10.1038/nature22963 28682339 PMC5830071

[14] Cheung KJ Padmanaban V Silvestri V: Polyclonal breast cancer metastases arise from collective dissemination of keratin 14-expressing tumor cell clusters. *Proc Natl Acad Sci U S A.* 2016;113(7):E854–63. 10.1073/pnas.1508541113 26831077 PMC4763783

[15] Wang H Yu C Gao X: The osteogenic niche promotes early-stage bone colonization of disseminated breast cancer cells. *Cancer Cell.* 2015;27(2):193–210. 10.1016/j.ccell.2014.11.017 25600338 PMC4326554

[16] Cheung KJ Gabrielson E Werb Z: Collective invasion in breast cancer requires a conserved basal epithelial program. *Cell.* 2013;155(7):1639–51. 10.1016/j.cell.2013.11.029 24332913 PMC3941206

[17] Campbell K: Contribution of epithelial-mesenchymal transitions to organogenesis and cancer metastasis. *Curr Opin Cell Biol.* 2018;55:30–5. 10.1016/j.ceb.2018.06.008 30006053 PMC6284102

[18] Lambert AW Pattabiraman DR Weinberg RA: Emerging Biological Principles of Metastasis. *Cell.* 2017;168(4):670–91. 10.1016/j.cell.2016.11.037 28187288 PMC5308465

[19] Grigore AD Jolly MK Jia D: Tumor Budding: The Name is EMT. Partial EMT. *J Clin Med.* 2016;5(5): pii: E51. 10.3390/jcm5050051 27136592 PMC4882480

[20] Zhang J Tian XJ Zhang H: TGF-β-induced epithelial-to-mesenchymal transition proceeds through stepwise activation of multiple feedback loops. *Sci Signal.* 2014;7(345):ra91. 10.1126/scisignal.2005304 25270257

[21] Huang RY Wong MK Tan TZ: An EMT spectrum defines an anoikis-resistant and spheroidogenic intermediate mesenchymal state that is sensitive to e-cadherin restoration by a src-kinase inhibitor, saracatinib (AZD0530). *Cell Death Dis.* 2013;4(11):e915. 10.1038/cddis.2013.442 24201814 PMC3847320

[22] Bierie B Pierce SE Kroeger C: Integrin-β4 identifies cancer stem cell-enriched populations of partially mesenchymal carcinoma cells. *Proc Natl Acad Sci U S A.* 2017;114(12):E2337–E2346. 10.1073/pnas.1618298114 28270621 PMC5373369

[23] Hong T Watanabe K Ta CH: An Ovol2-Zeb1 Mutual Inhibitory Circuit Governs Bidirectional and Multi-step Transition between Epithelial and Mesenchymal States. *PLoS Comput Biol.* 2015;11(11):e1004569. 10.1371/journal.pcbi.1004569 26554584 PMC4640575

[24] Jolly MK Tripathi SC Jia D: Stability of the hybrid epithelial/mesenchymal phenotype. *Oncotarget.* 2016;7(19):27067–84. 10.18632/oncotarget.8166 27008704 PMC5053633

[25] Pastushenko I Brisebarre A Sifrim A: Identification of the tumour transition states occurring during EMT. *Nature.* 2018;556(7702):463–8. 10.1038/s41586-018-0040-3 29670281

[26] Ruscetti M Quach B Dadashian EL: Tracking and Functional Characterization of Epithelial-Mesenchymal Transition and Mesenchymal Tumor Cells during Prostate Cancer Metastasis. *Cancer Res.* 2015;75(13):2749–59. 10.1158/0008-5472.CAN-14-3476 25948589 PMC4490048

[27] Cheung KJ Ewald AJ: A collective route to metastasis: Seeding by tumor cell clusters. *Science.* 2016;352(6282):167–9. 10.1126/science.aaf6546 27124449 PMC8183671

[28] Micalizzi DS Maheswaran S Haber DA: A conduit to metastasis: circulating tumor cell biology. *Genes Dev.* 2017;31(18):1827–40. 10.1101/gad.305805.117 29051388 PMC5695084

[29] Alix-Panabières C Pantel K: Challenges in circulating tumour cell research. *Nat Rev Cancer.* 2014;14(9):623–31. 10.1038/nrc3820 25154812

[30] Friedl P Locker J Sahai E: Classifying collective cancer cell invasion. *Nat Cell Biol.* 2012;14(8):777–83. 10.1038/ncb2548 22854810

[31] Aceto N Bardia A Miyamoto DT: Circulating tumor cell clusters are oligoclonal precursors of breast cancer metastasis. *Cell.* 2014;158(5):1110–22. 10.1016/j.cell.2014.07.013 25171411 PMC4149753

[32] Najor NA: Desmosomes in Human Disease. *Annu Rev Pathol.* 2018;13:51–70. 10.1146/annurev-pathol-020117-044030 29414250

[33] Aktary Z Alaee M Pasdar M: Beyond cell-cell adhesion: Plakoglobin and the regulation of tumorigenesis and metastasis. *Oncotarget.* 2017;8(19):32270–91. 10.18632/oncotarget.15650 28416759 PMC5458283

[34] Kai K Iwamoto T Zhang D: CSF-1/CSF-1R axis is associated with epithelial/mesenchymal hybrid phenotype in epithelial-like inflammatory breast cancer. *Sci Rep.* 2018;8(1): 9427. 10.1038/s41598-018-27409-x 29930294 PMC6013474

[35] Matsuzawa K Himoto T Mochizuki Y: α-Catenin Controls the Anisotropy of Force Distribution at Cell-Cell Junctions during Collective Cell Migration. *Cell Rep.* 2018;23(12):3447–56. 10.1016/j.celrep.2018.05.070 29924989

[36] Labernadie A Kato T Brugués A: A mechanically active heterotypic E-cadherin/N-cadherin adhesion enables fibroblasts to drive cancer cell invasion. *Nat Cell Biol.* 2017;19(3):224–37. 10.1038/ncb3478 28218910 PMC5831988

[37] Brabletz T Jung A Spaderna S: Opinion: migrating cancer stem cells - an integrated concept of malignant tumour progression. *Nat Rev Cancer.* 2005;5(9):744–9. 10.1038/nrc1694 16148886

[38] Mani SA Guo W Liao MJ: The epithelial-mesenchymal transition generates cells with properties of stem cells. *Cell.* 2008;133(4):704–15. 10.1016/j.cell.2008.03.027 18485877 PMC2728032

[39] Wellner U Schubert J Burk UC: The EMT-activator ZEB1 promotes tumorigenicity by repressing stemness-inhibiting microRNAs. *Nat Cell Biol.* 2009;11(12):1487–95. 10.1038/ncb1998 19935649

[40] Hwang WL Jiang JK Yang SH: MicroRNA-146a directs the symmetric division of Snail-dominant colorectal cancer stem cells. *Nat Cell Biol.* 2014;16(3):268–80. 10.1038/ncb2910 24561623

[41] Pattabiraman DR Weinberg RA: Targeting the Epithelial-to-Mesenchymal Transition: The Case for Differentiation-Based Therapy. *Cold Spring Harb Symp Quant Biol.* 2016;81:11–9. 10.1101/sqb.2016.81.030957 28057845 PMC5722631

[42] Pattabiraman DR Bierie B Kober KI: Activation of PKA leads to mesenchymal-to-epithelial transition and loss of tumor-initiating ability. *Science.* 2016;351(6277): aad3680. 10.1126/science.aad3680 26941323 PMC5131720

[43] Ocaña OH Córcoles R Fabra A: Metastatic colonization requires the repression of the epithelial-mesenchymal transition inducer Prrx1. *Cancer Cell.* 2012;22(6):709–24. 10.1016/j.ccr.2012.10.012 23201163

[44] Tsai JH Donaher JL Murphy DA: Spatiotemporal regulation of epithelial-mesenchymal transition is essential for squamous cell carcinoma metastasis. *Cancer Cell.* 2012;22(6):725–36. 10.1016/j.ccr.2012.09.022 23201165 PMC3522773

[45] Stankic M Pavlovic S Chin Y: TGF-β-Id1 signaling opposes Twist1 and promotes metastatic colonization via a mesenchymal-to-epithelial transition. *Cell Rep.* 2013;5(5):1228–42. 10.1016/j.celrep.2013.11.014 24332369 PMC3891470

[46] Schmidt JM Panzilius E Bartsch HS: Stem-cell-like properties and epithelial plasticity arise as stable traits after transient Twist1 activation. *Cell Rep.* 2015;10(2):131–9. 10.1016/j.celrep.2014.12.032 25578726

[47] Beck B Lapouge G Rorive S: Different levels of Twist1 regulate skin tumor initiation, stemness, and progression. *Cell Stem Cell.* 2015;16(1):67–79. 10.1016/j.stem.2014.12.002 25575080

[48] Grosse-Wilde A Fouquier d'Hérouël A McIntosh E: Stemness of the hybrid Epithelial/Mesenchymal State in Breast Cancer and Its Association with Poor Survival. *PLoS One.* 2015;10(5):e0126522. 10.1371/journal.pone.0126522 26020648 PMC4447403

[49] Reichert M Bakir B Moreira L: Regulation of Epithelial Plasticity Determines Metastatic Organotropism in Pancreatic Cancer. *Dev Cell.* 2018;45(6):696–711.e8. 10.1016/j.devcel.2018.05.025 29920275 PMC6011231

[50] Clevers H Loh KM Nusse R: Stem cell signaling. An integral program for tissue renewal and regeneration: Wnt signaling and stem cell control. *Science.* 2014;346(6205): 1248012. 10.1126/science.1248012 25278615

[51] Basu S Haase G Ben-Ze'ev A: Wnt signaling in cancer stem cells and colon cancer metastasis [version 1; referees: 3 approved]. *F1000Res.* 2016;5: pii: F1000 Faculty Rev-699. 10.12688/f1000research.7579.1 27134739 PMC4841194

[52] Zöller M: CD44: Can a cancer-initiating cell profit from an abundantly expressed molecule? *Nat Rev Cancer.* 2011;11(4):254–67. 10.1038/nrc3023 21390059

[53] Orian-Rousseau V Sleeman J: CD44 is a multidomain signaling platform that integrates extracellular matrix cues with growth factor and cytokine signals. *Adv Cancer Res.* 2014;123:231–54. 10.1016/B978-0-12-800092-2.00009-5 25081532

[54] Xu H Tian Y Yuan X: The role of CD44 in epithelial-mesenchymal transition and cancer development. *Onco Targets Ther.* 2015;8:3783–92. 10.2147/OTT.S95470 26719706 PMC4689260

[55] Schmitt M Metzger M Gradl D: CD44 functions in Wnt signaling by regulating LRP6 localization and activation. *Cell Death Differ.* 2015;22(4):677–89. 10.1038/cdd.2014.156 25301071 PMC4356338

[56] Yu H Lee H Herrmann A: Revisiting STAT3 signalling in cancer: New and unexpected biological functions. *Nat Rev Cancer.* 2014;14(11):736–46. 10.1038/nrc3818 25342631

[57] Lee JL Wang MJ Chen JY: Acetylation and activation of STAT3 mediated by nuclear translocation of CD44. *J Cell Biol.* 2009;185(6):949–57. 10.1083/jcb.200812060 19506034 PMC2711621

[58] Shen YA Wang CY Chuang HY: CD44 and CD24 coordinate the reprogramming of nasopharyngeal carcinoma cells towards a cancer stem cell phenotype through STAT3 activation. *Oncotarget.* 2016;7(36):58351–66. 10.18632/oncotarget.11113 27521216 PMC5295435

[59] Marotta LL Almendro V Marusyk A: The JAK2/STAT3 signaling pathway is required for growth of CD44⁺CD24⁻ stem cell-like breast cancer cells in human tumors. *J Clin Invest.* 2011;121(7):2723–35. 10.1172/JCI44745 21633165 PMC3223826

[60] Huang P Chen A He W: BMP-2 induces EMT and breast cancer stemness through Rb and CD44. *Cell Death Discov.* 2017;3: 17039. 10.1038/cddiscovery.2017.39 28725489 PMC5511860

[61] Yan Y Zuo X Wei D: Concise Review: Emerging Role of CD44 in Cancer Stem Cells: A Promising Biomarker and Therapeutic Target. *Stem Cells Transl Med.* 2015;4(9):1033–43. 10.5966/sctm.2015-0048 26136504 PMC4542874

[62] Gavert N Conacci-Sorrell M Gast D: L1, a novel target of beta-catenin signaling, transforms cells and is expressed at the invasive front of colon cancers. *J Cell Biol.* 2005;168(4):633–42. 10.1083/jcb.200408051 15716380 PMC2171754

[63] Gavert N Sheffer M Raveh S: Expression of L1-CAM and ADAM10 in human colon cancer cells induces metastasis. *Cancer Res.* 2007;67(16):7703–12. 10.1158/0008-5472.CAN-07-0991 17699774

[64] Ben-Shmuel A Shvab A Gavert N: Global analysis of L1-transcriptomes identified IGFBP-2 as a target of ezrin and NF-κB signaling that promotes colon cancer progression. *Oncogene.* 2013;32(27):3220–30. 10.1038/onc.2012.340 22869145

[65] Shvab A Haase G Ben-Shmuel A: Induction of the intestinal stem cell signature gene SMOC-2 is required for L1-mediated colon cancer progression. *Oncogene.* 2016;35(5):549–57. 10.1038/onc.2015.127 25915847

[66] Schwitalla S Fingerle AA Cammareri P: Intestinal tumorigenesis initiated by dedifferentiation and acquisition of stem-cell-like properties. *Cell.* 2013;152(1–2):25–38. 10.1016/j.cell.2012.12.012 23273993

[67] Basu S Gavert N Brabletz T Ben-Ze'ev A: The intestinal stem cell regulating gene ASCL2 is required for L1-mediated colon cancer progression. *Cancer Lett.* 2018;424:9–18. 10.1016/j.canlet.2018.03.022 29551399

[68] Shtutman M Levina E Ohouo P: Cell adhesion molecule L1 disrupts E-cadherin-containing adherens junctions and increases scattering and motility of MCF7 breast carcinoma cells. *Cancer Res.* 2006;66(23):11370–80. 10.1158/0008-5472.CAN-06-2106 17145883

[69] Schuijers J Junker JP Mokry M: Ascl2 acts as an R-spondin/Wnt-responsive switch to control stemness in intestinal crypts. *Cell Stem Cell.* 2015;16(2):158–70. 10.1016/j.stem.2014.12.006 25620640

[70] Colombo F Meldolesi J: L1-CAM and N-CAM: From Adhesion Proteins to Pharmacological Targets. *Trends Pharmacol Sci.* 2015;36(11):769–81. 10.1016/j.tips.2015.08.004 26478212

[71] Taddei A Giampietro C Conti A: Endothelial adherens junctions control tight junctions by VE-cadherin-mediated upregulation of claudin-5. *Nat Cell Biol.* 2008;10(8):923–34. 10.1038/ncb1752 18604199

[72] Rognoni E Widmaier M Jakobson M: Kindlin-1 controls Wnt and TGF-β availability to regulate cutaneous stem cell proliferation. *Nat Med.* 2014;20(4):350–9. 10.1038/nm.3490 24681597 PMC3982140

[73] Desgrosellier JS Lesperance J Seguin L: Integrin αvβ3 drives slug activation and stemness in the pregnant and neoplastic mammary gland. *Dev Cell.* 2014;30(3):295–308. 10.1016/j.devcel.2014.06.005 25117682 PMC4147869

[74] Visvader JE: Keeping abreast of the mammary epithelial hierarchy and breast tumorigenesis. *Genes Dev.* 2009;23(22):2563–77. 10.1101/gad.1849509 19933147 PMC2779757

[75] Medema JP: Cancer stem cells: the challenges ahead. *Nat Cell Biol.* 2013;15(4):338–44. 10.1038/ncb2717 23548926

[76] Lathia JD Gallagher J Heddleston JM: Integrin alpha 6 regulates glioblastoma stem cells. *Cell Stem Cell.* 2010;6(5):421–32. 10.1016/j.stem.2010.02.018 20452317 PMC2884275

[77] Seguin L Kato S Franovic A: An integrin β _3_-KRAS-RalB complex drives tumour stemness and resistance to EGFR inhibition. *Nat Cell Biol.* 2014;16(5):457–68. 10.1038/ncb2953 24747441 PMC4105198

[78] Seguin L Desgrosellier JS Weis SM: Integrins and cancer: Regulators of cancer stemness, metastasis, and drug resistance. *Trends Cell Biol.* 2015;25(4):234–40. 10.1016/j.tcb.2014.12.006 25572304 PMC4380531

[79] Hamidi H Ivaska J: Every step of the way: integrins in cancer progression and metastasis. *Nat Rev Cancer.* 2018;18(9):533–548. 10.1038/s41568-018-0038-z 30002479 PMC6629548

[80] Wiesner S Legate KR Fässler R: Integrin-actin interactions. *Cell Mol Life Sci.* 2005;62(10):1081–99. 10.1007/s00018-005-4522-8 15761669 PMC11139080

[81] Hannigan G Troussard AA Dedhar S: Integrin-linked kinase: A cancer therapeutic target unique among its ILK. *Nat Rev Cancer.* 2005;5(1):51–63. 10.1038/nrc1524 15630415

[82] Tsoumas D Nikou S Giannopoulou E: ILK expression in colorectal cancer is associated with EMT, cancer stem cell markers and chemoresistance. *Cancer Genomics Proteomics.* 2018;15(2):127–41. 10.21873/cgp.20071 29496692 PMC5892607

[83] Tan C Costello P Sanghera J: Inhibition of integrin linked kinase (ILK) suppresses beta-catenin-Lef/Tcf-dependent transcription and expression of the E-cadherin repressor, snail, in APC-/- human colon carcinoma cells. *Oncogene.* 2001;20(1):133–40. 10.1038/sj.onc.1204052 11244511

[84] Marotta A Tan C Gray V: Dysregulation of integrin-linked kinase (ILK) signaling in colonic polyposis. *Oncogene.* 2001;20(43):6250–7. 10.1038/sj.onc.1204791 11593435

[85] Bravou V Klironomos G Papadaki E: ILK over-expression in human colon cancer progression correlates with activation of beta-catenin, down-regulation of E-cadherin and activation of the Akt-FKHR pathway. *J Pathol.* 2006;208(1):91–9. 10.1002/path.1860 16278819

[86] Bravou V Klironomos G Papadaki E: Integrin-linked kinase (ILK) expression in human colon cancer. *Br J Cancer.* 2003;89(12):2340–1. 10.1038/sj.bjc.6601482 14676816 PMC2395290

[87] Novak A Hsu SC Leung-Hagesteijn C: Cell adhesion and the integrin-linked kinase regulate the LEF-1 and beta-catenin signaling pathways. *Proc Natl Acad Sci U S A.* 1998;95(8):4374–9. 10.1073/pnas.95.8.4374 9539744 PMC22496

[88] Oloumi A McPhee T Dedhar S: Regulation of E-cadherin expression and β-catenin/Tcf transcriptional activity by the integrin-linked kinase. *Biochim Biophys Acta.* 2004;1691(1):1–15. 10.1016/j.bbamcr.2003.12.002 15053919

[89] Hsu EC Kulp SK Huang HL: Integrin-linked kinase as a novel molecular switch of the IL-6-NF-κB signaling loop in breast cancer. *Carcinogenesis.* 2016;37(4):430–42. 10.1093/carcin/bgw020 26905583 PMC5006214

[90] Hsu EC Kulp SK Huang HL: Function of integrin-linked kinase in modulating the stemness of IL-6-abundant breast cancer cells by regulating γ-secretase-mediated Notch1 activation in caveolae. *Neoplasia.* 2015;17(6):497–508. 10.1016/j.neo.2015.06.001 26152358 PMC4719004

[91] Pang MF Siedlik MJ Han S: Tissue stiffness and hypoxia modulate the integrin-linked kinase ILK to control breast cancer stem-like cells. *Cancer Res.* 2016;76(18):5277–87. 10.1158/0008-5472.CAN-16-0579 27503933 PMC5026611

